# PARP Inhibitors in Clinical Use Induce Genomic Instability in Normal Human Cells

**DOI:** 10.1371/journal.pone.0159341

**Published:** 2016-07-18

**Authors:** Shuhei Ito, Conleth G. Murphy, Ekaterina Doubrovina, Maria Jasin, Mary Ellen Moynahan

**Affiliations:** 1 Developmental Biology Program, Memorial Sloan Kettering Cancer Center, New York, New York, United States of America; 2 Department of Medicine, Memorial Sloan Kettering Cancer Center, New York, New York, United States of America; 3 Department of Pediatrics, Memorial Sloan Kettering Cancer Center, New York, New York, United States of America; 4 Department of Surgery and Science, Graduate School of Medical Sciences, Kyushu University, Higashi-ku, Fukuoka, Japan; Tel-Aviv University, ISRAEL

## Abstract

Poly(ADP-ribose) polymerases (PARPs) are the first proteins involved in cellular DNA repair pathways to be targeted by specific inhibitors for clinical benefit. Tumors harboring genetic defects in homologous recombination (HR), a DNA double-strand break (DSB) repair pathway, are hypersensitive to PARP inhibitors (PARPi). Early phase clinical trials with PARPi have been promising in patients with advanced BRCA1 or BRCA2-associated breast, ovary and prostate cancer and have led to limited approval for treatment of BRCA-deficient ovary cancer. Unlike HR-defective cells, HR-proficient cells manifest very low cytotoxicity when exposed to PARPi, although they mount a DNA damage response. However, the genotoxic effects on normal human cells when agents including PARPi disturb proficient cellular repair processes have not been substantially investigated. We quantified cytogenetic alterations of human cells, including primary lymphoid cells and non-tumorigenic and tumorigenic epithelial cell lines, exposed to PARPi at clinically relevant doses by both sister chromatid exchange (SCE) assays and chromosome spreading. As expected, both olaparib and veliparib effectively inhibited poly-ADP-ribosylation (PAR), and caused marked hypersensitivity in HR-deficient cells. Significant dose-dependent increases in SCEs were observed in normal and non-tumorigenic cells with minimal residual PAR activity. Clinically relevant doses of the FDA-approved olaparib led to a marked increase of SCEs (5-10-fold) and chromatid aberrations (2-6-fold). Furthermore, olaparib potentiated SCE induction by cisplatin in normal human cells. Our data have important implications for therapies with regard to sustained genotoxicity to normal cells. Genomic instability arising from PARPi warrants consideration, especially if these agents will be used in people with early stage cancers, in prevention strategies or for non-oncologic indications.

## Introduction

Poly(ADP-ribose) polymerase 1 (PARP1) and PARP2 are rapidly activated by DNA strand breaks where through target-protein ribosylation they promote the repair of DNA single-strand breaks (SSBs) and coordinate cellular responses to stress [1,2]. In cellular extracts, poly-ADP-ribosylation (PAR) stimulated by DNA strand breaks is primarily mediated by PARP1, while PARP2 is responsible for 10–15% of the total activity [3]. Mice deficient for PARP1 or PARP2 are hypersensitive to γ-irradiation and alkylating agents, and demonstrate increased genomic instability with elevated sister chromatid exchanges (SCEs) [4–8], suggesting that neither PARP1 nor PARP2 alone can compensate completely for the loss of the other. *Parp1*^*-/-*^ and *Parp2*^*-/-*^ mice are viable [6,7], however, *Parp1*^*-/-*^ *Parp2*^*-/-*^ double mutant mice die early in embryogenesis, demonstrating the essential requirement for nuclear PAR [7]. Significantly, PARP1 deficiency alone induces mammary carcinoma and tumorigenesis is markedly increased when combined with other DNA damage response (DDR) genetic deficiencies [9]. Notably, less potent PARP inhibitors (PARPi), which did not demonstrate synthetic lethality in BRCA-deficient tumor cells, had been shown to increase SCEs 2-fold in normal human cells at high doses [10–12].

Highly potent PARP inhibitors (PARPi) have been developed for clinical use in cancer therapy. PARPi monotherapy has demonstrated clinical activity in tumors lacking BRCA1 and BRCA2 function where enhancement of the DNA repair defect led to tumor reduction [13–16]. A large randomized trial in recurrent ovarian cancer demonstrated improved progression-free survival in patients taking olaparib, leading to the approval of olaparib treatment for BRCA-associated recurrent ovary cancer in both Europe and the United States [17]. However, these results were not defined as a success in Israel, where PARPi have not been approved for treatment of BRCA-associated ovary cancer, yet the prevalence of women harboring BRCA mutations is higher than in other countries.

Currently, trials with PARPi are underway in early stage breast cancer, and are under consideration as a prevention strategy in *BRCA*1 and *BRCA*2 mutation carriers and for an array of non-oncologic conditions that stem from its role in inhibition of ADP-ribosylation of other PARP family members including tankyrases, ATP/NAD depletion and transcriptional regulation [18–20]. Although well tolerated from an overt toxicity profile, phosphorylated histone H2AX (γH2AX) foci are observed following PARPi treatment to the same extent in wild-type and BRCA1/2-deficient cell lines as well as in hair follicles from patients, suggesting that DNA double-strand breaks (DSBs) are incurred independent of BRCA function [13,21]. This finding indicates that inhibiting nuclear PAR in proliferating cells by PARPi may lead to the accumulation of DNA damage even in repair-proficient cells and raises a concern for sustained genotoxicity.

The clinical use of PARPi together with the phenotypes associated with genetic PARP deficiency—genomic instability observed in single knockout mice, tumorigenesis markedly enhanced by loss of DNA damage signaling proteins and the non-viability of mice without nuclear ADP-ribosylation function—led us to assess the genomic instability incurred in human repair-proficient cells following chemical inhibition of PARP activity.

Sister chromatid exchange (SCE) is a recombination event between identical sister chromatids following DNA replication, typically but not uniformly, resulting in an equal exchange of genetic information. However, SCEs that occur at offset repeats can lead to loss or gain of genetic information. An assay using differential staining of the chromatids based on the timing of newly replicated DNA has been used to quantify the number of SCEs observed at metaphase [22,23]. Increased SCEs are thus a measure of hyperrecombination leading to chromatid exchange, whether due to deficiencies in genes involved in the suppression of exchange such as the BLM helicase, loss of which leads to the pan cancer-prone Bloom syndrome, or due to unrepaired DNA damage encountered during DNA replication and subsequent HR-mediated repair at stalled replication forks [24–26]. An increase in SCEs correlates with mutagenic potential [27] and induced loss of heterozygosity (LOH) [28–30].

In this work, we examined the frequency of SCE induction in human cells treated with PARPi currently in clinical use, olaparib and veliparib, and as a control, an earlier agent purported to be a PARPi (BSI-201) [31]. Effective PARP inhibition with olaparib and veliparib induce genomic instability in all human cells examined, resulting in a marked increase of SCE frequencies and chromatid-type aberrations. These data challenge the current view that repair-proficient cells are unaffected by PARP inhibition.

## Results

### The PARPi olaparib increases SCE frequencies in repair-proficient human cells

To ascertain whether genotoxicity is incurred in repair proficient human cells with inhibition of PARP activity, we used a variety of cell types: breast epithelial cell lines, both non-transformed (MCF-10A, HMEC-hTERT) and tumorigenic (MDA-MB-468, MCF-7), an EBV-transformed B lymphocyte cell line (EBV-BL), and primary T cells from two individuals. The PARPi investigated was olaparib, which is highly inhibitory to both PARP1 and PARP2 [32]. As a prelude to determining SCE frequency, doubling time was determined for each cell type in the absence of olaparib (S1A Fig, S1 Table). Olaparib cytotoxicity upon continuous exposure with clinically achievable doses was also determined (S1B Fig). The IC_50s_ with continuous exposure were typically >2 μM, such that 1 μM was a sublethal dose considered to be suitable for SCE analysis for all cell types over the short-term of the experiment (two cell cycles).

**Fig 1:**
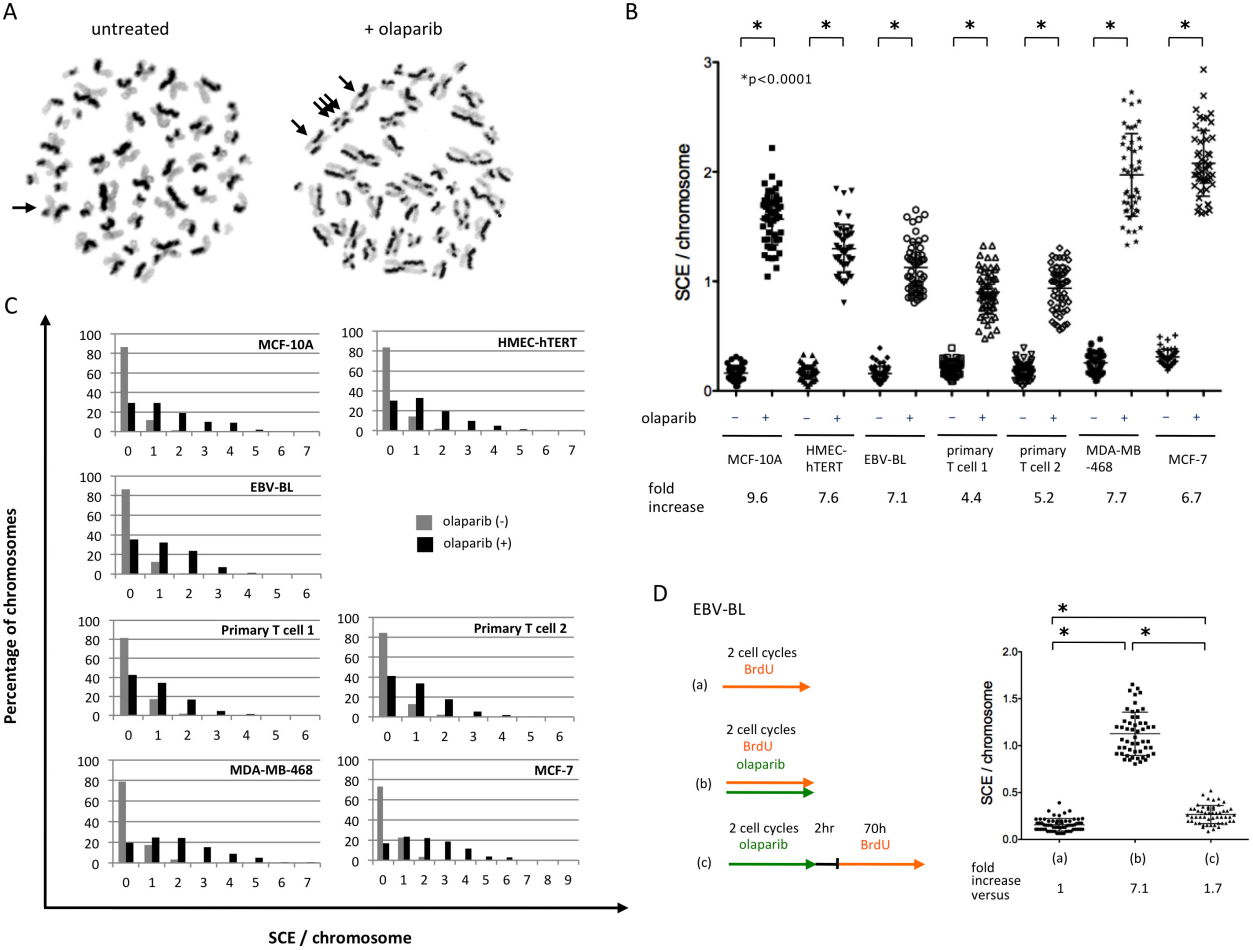
Olaparib increases the SCE frequency in repair-proficient human cells. **A.** Cells were exposed with BrdU and 1μM olaparib during two cell cycle periods. Representatives of two metaphase spreads harvested from untreated (left) and olaparib treated (right) primary T cells 2. Arrows, e.g. site of SCE. **B.** Spontaneous and olaparib-induced SCEs by cell type. The y-axis is the number of SCE per chromosome for each metaphase counted. Approximately 50 metaphases were counted for each cell type. In order to compare each cell type, the number of SCEs per metaphase was divided by chromosome number. Fold increases of SCE per chromosome by olaparib for each cell type are shown. Error bars identify the mean with SD. Asterisks designate statistical significance at *P* < 0.0001 using unpaired t-test. **C.** Multiple SCEs per chromosome are observed following olaparib. Histograms show the percentage of chromosomes categorized by number of SCEs per chromosome by cell type and exposure. **D.** Temporal induction of SCEs by olaparib. (a) Spontaneous SCE, vehicle treated EBV-BL cells (90 hr—BrdU). (b) Acute olaparib-induced SCE (1μM olaparib and BrdU). (c) Olaparib-exposure (1μM) followed by removal and 2 cell cycle BrdU exposure. Fold increases of SCE per chromosome versus (a) of each exposure are shown. Error bars identify the mean with SD. Asterisks designate statistical significance at *P* < 0.0001 using unpaired t-test.

The average number of SCE events in untreated human cells depicted by differential BrdU staining of sister chromatids is ~7 with a range of 1 to 14 [33]. The SCE frequency for the untreated non-tumorigenic cell lines and primary cells was 7.3 to 9.5 SCEs per metaphase, while that for the two tumorigenic cell lines was significantly higher with 13.9 and 22.7 SCEs per metaphase (S2A Fig, S2 Table). Thus, the majority of chromosomes of untreated cells had not incurred an SCE, and those that had, usually incurred a single SCE (Fig 1A–1C). With exposure to olaparib, SCEs were significantly induced in all cell types by a factor of 4.4 to 9.6 (Fig 1A–1C; S2 Table). Notably, multiple SCEs/chromosome were frequently observed with PARP inhibition, such that chromosomes without an SCE were in a minority (Fig 1C). The markedly elevated SCE frequency indicates that olaparib exposure in normally proliferating cells may cause genome destabilizing events through hyperrecombination.

### SCE frequencies decrease after olaparib removal

PARP1 and PARP2 rapidly respond to DNA damage, such that within seconds they recognize DNA strand breaks to initiate a cellular response to damage. The proteins are then rapidly metabolized by PARG, poly-ADP-ribose glycohydrolase, to control the extent and duration of target protein parylation [34]. Given the rapid kinetics of PARP regulation and the generally rapid repair of DNA SSBs, we hypothesized that the marked SCE induction would occur only in the presence of the PARPi. This is in contrast to DNA damaging agents that cause structural DNA alterations where SCE elevations can be more persistent [35]. To test this, EBV-BLs were pre-treated with olaparib for two cell cycles prior to incubation with BrdU rather than at the same time (Fig 1D). Although the SCE frequency of olaparib pre-treated cells was still significantly higher than that of untreated cells, the marked SCE induction observed in the presence of olaparib was substantially attenuated (1.7-fold vs 7.1-fold).

### Olaparib increases chromatid-type aberrations in repair-proficient human cells

**Fig 2:**
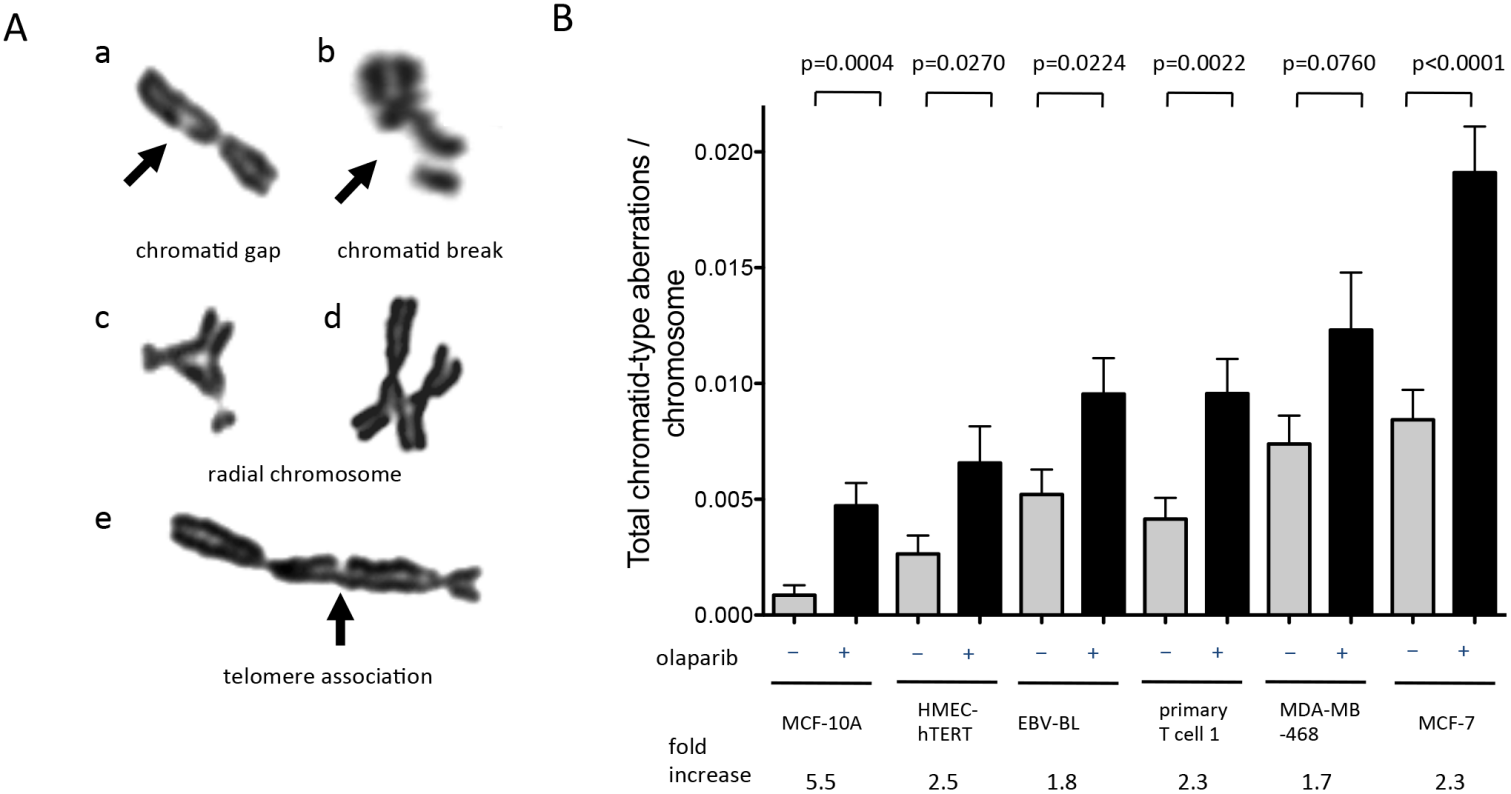
Olaparib increases chromatid-type aberrations in repair-proficient human cells. **A.** Representative chromatid-type aberrations in human cells include chromatid gap (a), chromatid break (b), radial chromosome (c and d) and telomere association (e). **B.** Olaparib-induced chromatid-type aberrations. Cells were exposed to vehicle or 1μM olaparib for 24 hrs. For each cell type, 100 metaphases were counted and the number of chromatid-type aberrations per metaphase was divided by chromosome number. Bars indicate the mean with SEM. The *P*-values were calculated using unpaired t-test.

Although SCEs at the identical location on both sister chromatids are genetically neutral, SCEs involving offset repeats on sister chromatids can lead to genetic loss or gain [36]. Importantly, elevated SCE frequencies are typically indicative of a capacity to incur additional genome-destabilizing events [37]. To determine if genomic instability is associated with PARP inhibition, we examined chromosomal aberrations after 24 h exposure to 1μM olaparib. We focused on chromatid-type aberrations because they are an indicator of the ongoing production of a primary structural change. Olaparib induced chromatid-type aberrations included gaps, breaks, radial chromosomes and telomere associations (Fig 2A). The average fold-increase in chromatid-type aberrations was 2.7 with a range of 1.7 to 5.5, which was significant in all cell types with the exception of MDA-MB-468 (p = 0.076) (Fig 2B; S3 Table). These results indicate that olaparib exposure acutely increases chromatid-type aberrations, an unequivocal indicator of compromised genomic integrity.

### Induction of SCE by PARPi is dose-dependent occurring at non-cytotoxic doses and associates with inhibition of PARP activity

**Fig 3:**
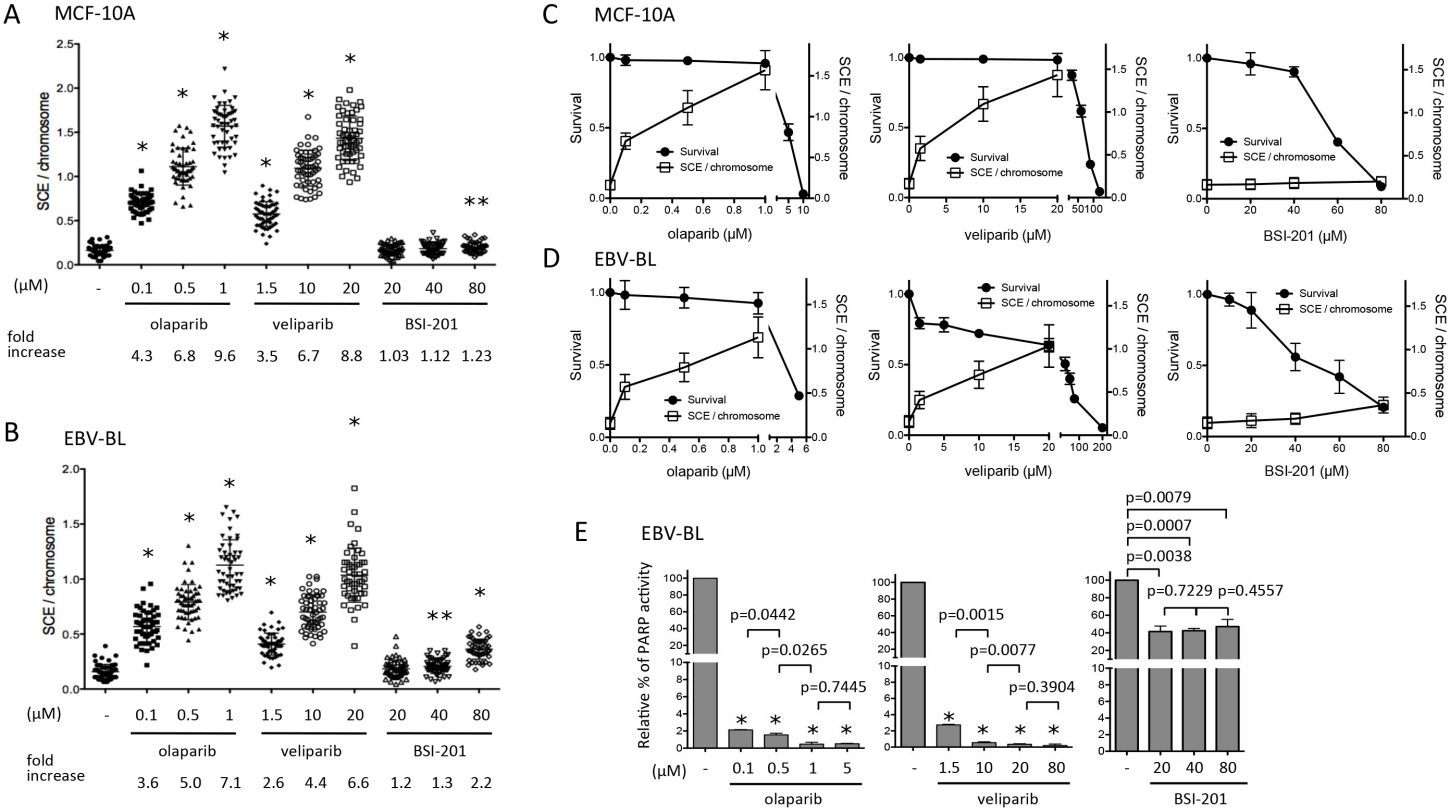
SCE induction increases with dose and associates with inhibition of PARP activity. Induction of SCEs occurs with increasing dose of olaparib and veliparib but not BSI-201in MCF-10A (A) and EBV-BL (B) cells. Approximately 50 metaphases were counted per cell type. Fold increases of SCE per chromosome are shown compared to vehicle treated cells. Error bars depict mean with SD. One and two asterisks designate statistical significance compared with vehicle treated cells at *P* < 0.0001 and *P* < 0.001, respectively. The *P*-values were calculated using unpaired t-test. Dose response curves for cell survival are depicted on the left y-axis (solid circles) with corresponding SCE frequency on the right y-axis for MCF-10A (C) and EBV-BL (D) cells with olaparib, veliparib and BSI-201. Means with SD represent 3 survival assays per cell type. The IC_50_ for olaparib, veliparib and BSI-201 were 4.7, 69.1 and 56.0 μM for MCF-10A, and 3.7, 42.5 and 48.9 μM for EBV-transformed B cells, respectively. **E.** Inhibition of cellular PARP activity in EBV-BL. Cells were incubated with or without increasing concentrations of olaparib, veliparib and BSI-201 24 hr before PARP activity was measured and values were normalized using protein concentration. Bars depict mean with SD of PARP activity for each concentration (μM) (n = 3). One asterisk designate statistical significance compared with untreated cells for paired *t*-test at *P* < 0.0001.

The large increase in SCEs with olaparib treatment led us to conduct dose-response experiments with olaparib, and a second PARPi in clinical use, veliparib, as well as BSI-201, a purported PARPi, in both MCF-10A and EBV-BL cells. Olaparib and veliparib induced a dose-dependent increase in the frequency of SCEs in both cell types at doses that were below the IC_10_, whereas BSI-201 resulted in a minor increase and only at a markedly cytotoxic dose (Fig 3A–3D). Notably, the survival of MCF-10A and EBV-BL cells was 95.7 ± 1.9% and 92.6 ± 7.4%, respectively (mean ± SD; 1 μM, Fig 3C and 3D), while a 10-fold lower dose (0.1 μM) induced SCEs ~4-fold (Fig 3A and 3B). On the contrary, SCEs were not induced at non-cytotoxic doses of BSI-201.

To determine if SCE induction associates with the inhibition of cellular PARP activity following treatment with PARPi, we measured PAR levels in cell extracts. PARP activity decreased ~98% at the lowest doses of olaparib and veliparib tested in treated EBV-BLs as compared with that of untreated cells (Fig 3E). A further reduction to >99% decreased PARP activity was observed with the higher concentrations of olaparib and veliparib (Fig 3E). Most notably, SCEs significantly increased with the more complete reduction of PARP activity. Consistent with more recent reports questioning its effectiveness [31], PARP activity of BSI-201 treated EBV-BLs decreased modestly, by ~ 60% as compared with that of untreated cells. Thus, SCEs are induced at low doses of olaparib and veliparib and are further increased without an increase in cytotoxicity as residual PARP activity is inhibited.

### BRCA1- and BRCA2-deficient mouse cells are hypersensitive to olaparib and veliparib but not BSI-201

**Fig 4:**
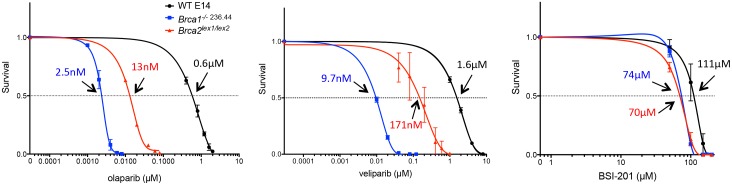
BRCA1- and BRCA2-deficient mES cells are hypersensitive to olaparib and veliparib but not BSI-201. The clonogenic survival assays of wild-type, *Brca1*^-/- 236.44^ and *Brca2*^*lex1/lex2*^ mES cells following treatment with olaparib, veliparib and BSI-201. BRCA1- and BRCA2-deficient cells were extremely sensitive to olaparib and veliparib as compared to wild-type cells. The concentration and arrows indicate the IC_50_ for each cell line. Error bars depict mean with SD (n = 3).

To correlate the relative activity of the tested PARP inhibitors with the paradigm of synthetic lethality in HR-defective cells, we determined the survival of *Brca1*^-/- 236.44^ and *Brca2*^*lex1/lex2*^ mouse embryonic stem (mES) cells following treatment with olaparib, veliparib or BSI-201. Consistent with previous reports which showed marked hypersensitivity of BRCA-deficient mES cells with PARP inhibitors KU0058684 and KU0058948 [21], clonogenic survival assays showed that BRCA1- and BRCA2-deficient cells were extremely sensitive to olaparib and veliparib as compared to wild-type cells (Fig 4). The IC_50_ for olaparib was 2.5 nM for *Brca1*^-/- 236.44^ cells and 13 nM for *Brca2*^*lex1/lex2*^ cells, but 600 nM for wild-type cells, indicating 240- and 46-fold enhanced sensitivity of cells lacking wild-type BRCA1 and BRCA2, respectively. Similar levels of enhanced sensitivity were found with veliparib. However, with BSI-201, the IC_50_ was 74 μM for *Brca1*^-/- 236.44^ cells and 70 μM for *Brca2*^*lex1/lex2*^ cells versus 111 μM for wild-type cells, indicating similar marginal increases of 1.5 and 1.6-fold, respectively, as compared with wild-type-cells. Thus, the cytotoxic effect of BSI-201 to HR-deficient cells is modest, shows little relationship to the HR repair defect, and is not dependent on PARP activity.

### Combined treatment with cisplatin and olaparib are additive in inducing genome destabilizing events

**Fig 5:**
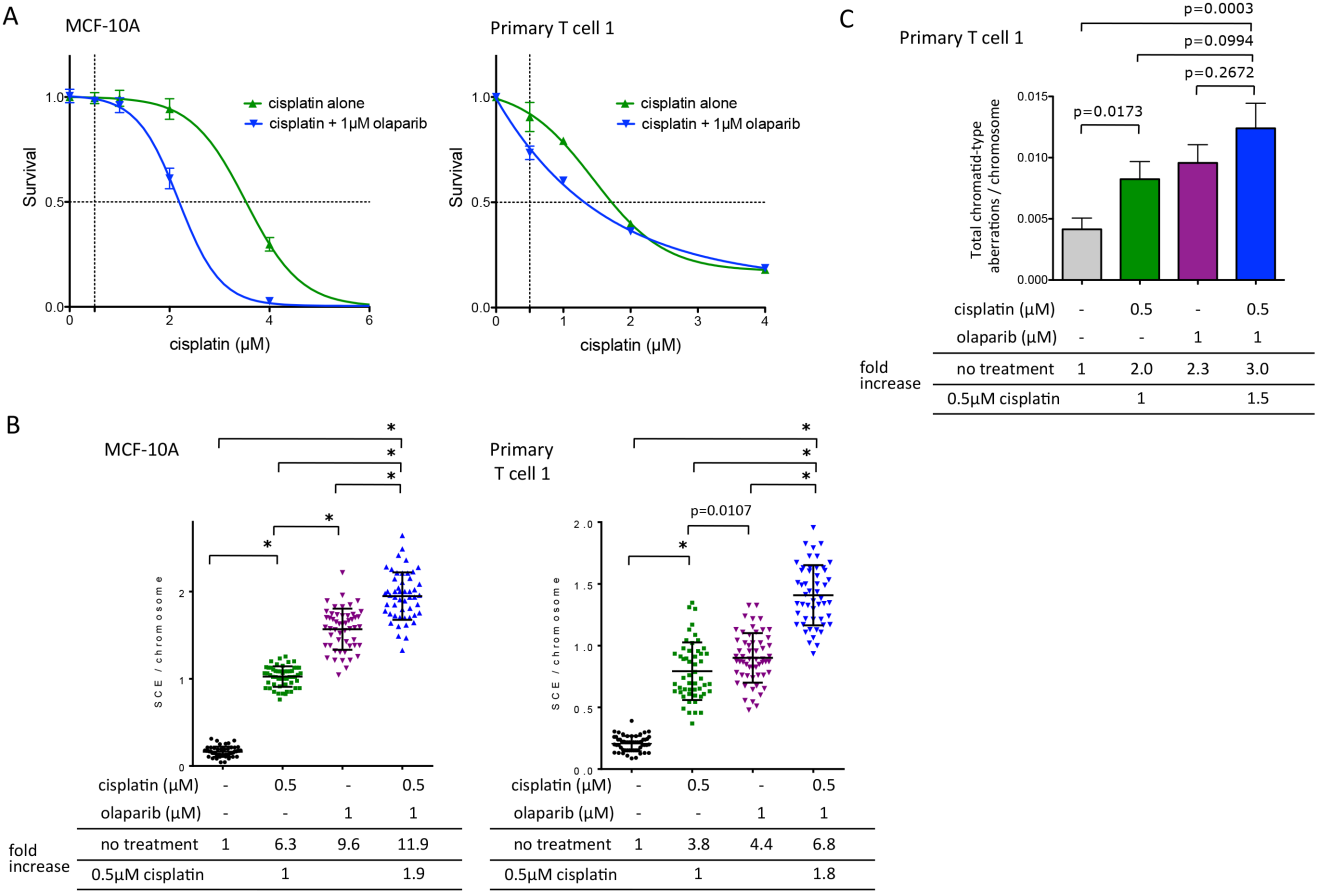
Potentiation of genomic instability with combined exposure of olaparib and cisplatin. **A.** Dose-response curve of survival with cisplatin alone and cisplatin with 1μM olaparib of MCF-10A and primary T cell 1. In MCF-10A, cells were grown with indicated concentration of cisplatin for the first 30 hr with or without continuous exposure at 1 μM olaparib. Clonogenic survival was assessed by colony counting at 9 days. In primary T cell 1, cells were grown with indicated concentration of cisplatin with or without 1 μM olaparib for the first 24 hr. Viable cells were scored and enumerated by trypan blue exclusion at 7 days. Means with SD are shown (MCF-10A: n = 3, primary T cell 1: n = 2). **B.** Induction of SCEs by cisplatin and/or olaparib in MCF-10A and primary T cell 1. In MCF-10A, cells were exposed at 0.5 μM cisplatin with or without 1 μM olaparib for 30 hr. In primary T cell 1, cells were exposed at 0.5 μM cisplatin for the first 24hr with or without 1 μM olaparib for 70 hr. Fold increases of SCE per chromosome compared with no treatment or 0.5 μM cisplatin are shown for each cell type. Approximately 50 metaphases were counted for each cell. Error bars depict the mean with SD. Asterisks designate statistical significance for unpaired *t*-test at *P* < 0.0001. **C.** Chromatid-type aberrations following cisplatin with or without olaparib in primary T cell 1. Cells were exposed at 0.5 μM cisplatin with or without 1 μM olaparib for 24 hr. One hundred metaphases were counted for each exposure. Fold increases of chromatid-type aberrations per chromosome compared with no treatment or 0.5 μM cisplatin are shown for each cell type. The y-axis is the number of total chromatid-type aberrations per chromosome for each metaphase counted. Error bars indicate mean with SEM. The *P*-values were calculated using unpaired t-test.

In HR-deficient tumors, the “synthetic lethality” strategy for PARP inhibition is dependent on unrepaired endogenous strand breaks that arise during DNA replication and normal cell metabolism. Clinical trials with combined therapy using both chemotherapy and PARPi are ongoing [18,38]. The strategy is to directly induce DNA damage with chemotherapy whilst also inhibiting SSB repair with PARP inhibition. To ascertain whether genotoxicity is further potentiated with PARPi during DNA damage-inducing chemotherapy, we treated cells with both cisplatin and olaparib and compared the SCE frequency to either agent alone. Dose response curves for cisplatin alone and cisplatin with 1 μM olaparib were established using MCF-10A and primary T cells (Fig 5A) to ensure that exposures to assess genome instability were performed at minimally cytotoxic doses. In MCF-10A cells, the IC_50_ was 3.4 μM for cisplatin alone and 2.4 μM for cisplatin in combination with 1 μM olaparib. In primary T cells, the IC_50_ was 1.74 μM for cisplatin alone and 1.43 μM for cisplatin in combination with 1 μM olaparib. Cells exposed to 0.5 μM cisplatin alone demonstrated a significant increase in SCEs (Fig 5B, S5 Table), with fold increases of 6.3 for MCF-10A cells and 3.8 for primary T cells as compared to untreated cells. The SCE induction following this low dose of cisplatin was significantly less than that seen with olaparib in the MCF-10A cells, although in primary T cells it was more comparable. Notably, the combination of 1 μM olaparib and 0.5 μM cisplatin resulted in significant SCE increases of 1.8–1.9-fold in both cell types as compared with 0.5 μM cisplatin alone. Combined therapy resulted in fold increases of 11.9 for MCF-10A and 6.8 for primary T cells as compared with untreated cells. In addition to the significant increase in SCEs, chromatid-type aberrations were also increased by combined therapy. Aberrations increased 2-fold in primary T cells with cisplatin alone and were increased another 1.5-fold with olaparib treatment, although this did not reach statistical significance (Fig 5C, S6 Table). These results provide evidence that olaparib potentiates the genomic instability of traditional DNA damaging agents, such as cisplatin, at non-cytotoxic concentrations.

## Discussion

Harnessing strategies to improve clinical outcomes in cancer therapy must generally be tempered by acute toxicity to normal tissues. Exploiting genetic differences in tumor cells as compared to normal cells has the potential to markedly enhance the therapeutic index. One such example is the marked cytotoxicity of HR-deficient cells to PARPi as compared to minimal cytotoxicity in repair-proficient cells. However, a potential concern is that disturbing DNA repair processes even in a repair-proficient setting may give rise to genetic instability. For this reason, we quantified PARPi-induced genotoxicity with the agents in current clinical use.

We performed this analysis using two cytogenetic biomarkers, SCE and chromatid-type aberrations, to ascertain single cell chromosome alterations following brief, low dose exposures. Increased genotoxicity induced by the PARPi olaparib was observed in all human cells analyzed, including primary cells and cell lines, lymphocytes and epithelial cells, and non-tumorigenic and tumorigenic cells. Following brief olaparib exposure, an average of ~40 SCEs per metaphase was observed in the primary T cells and an even greater number in the epithelial cell lines, such that a 4.4 to 9.6-fold increase in SCEs was observed compared with untreated cells.

This large number of SCEs observed following PARPi exposure is similar to that reported for Bloom syndrome [33], an inherited, cancer-prone disorder. Bloom syndrome is caused by defects in the BLM DNA helicase, which “dissolves” recombination intermediates, such that loss of BLM results in a hyperrecombination phenotype and genomic instability, including radial chromosome formation [39]. Individuals with this disorder develop a broad spectrum of tumors at an early age and often develop multiple tumors, including leukemias, lymphomas, and solid tumors.

The increase in SCEs observed with cisplatin was significant at low dose but less than that induced by olaparib and additive when both were used in conjunction. These data suggest that olaparib induces and enhances genotoxicity in a similar range to that of a known DNA damaging anticancer agent. A clinical concern is that the PARPi in current use are delivered on a continuous, twice daily dosing schedule as compared to standard chemotherapy administered intermittently [40]. Thus, the near continuous exposure to PARPi has the potential to cause genome instability in all proliferating cells.

Elevated SCE frequency is found to associate with genome destabilizing events, including the propensity to mutations and LOH, while unequal SCE events can result in gene dosage imbalance [37]. As a corollary to the markedly elevated SCE frequency, we identified olaparib-induced chromatid-type aberrations to be 1.7 to 5.5-fold higher as compared to untreated cells. Notably, 12 radial chromosomes were identified in olaparib-treated cells, whereas only 1 radial chromosome was observed in an untreated cancer cell. The increase in chromosome aberrations is of concern especially in light of the considered use of these agents in prevention and early stage cancer and non-oncologic indications [18,19].

γH2AX foci and PAR levels were reported as pharmacodynamic biomarkers to measure DNA damage during patient treatment [41]; thus it is known that the DNA damage response is activated in repair-proficient cells upon PARPi exposure. We found that genomic instability incurred with low dose olaparib and veliparib was associated with highly effective inhibition of PARP activity. This suggests that even very low doses of PARPi results in persistent unrepaired DNA in actively proliferating repair-proficient cells. The hyperrecombination we observed with the elevated SCE frequency is consistent with unresolved breaks persisting and disturbing normal DNA replication. An SCE can be considered the cell’s “next best” recourse when presented with DNA damage at a stalled replication fork. Yet, the increase in chromatid-type aberrations confirms that the increase in SCEs does not fully succeed in rescuing all the DNA breaks occurring with PARP inhibition. The cellular fate of chromosome structural aberrations and more subtle gene dosage disturbances due to unequal SCEs will be variable, ranging from no deleterious effect, to a genetically-induced growth advantage, to cell elimination. Gene dosage alterations resulting from unequal SCE or LOH events will be locus specific, whereas a cell harboring a radial chromosome would be expected to undergo elimination. This may manifest in reduced self renewal capacity in certain organs and/or an accelerating aging phenotype. Successful propagation of structural aberrations in cells with defective checkpoints may promote tumorigenesis.

Clinical evidence supporting relevant levels of genotoxicity associated with PARPi is the 2.2% incidence of myelodysplastic syndrome and acute myelogenous leukemia in patients on the olaparib-treated arm of the Phase III advanced ovary cancer trial, as reported to the FDA Oncology Drugs Advisory Committee [42]. These patients had all received prior cytotoxic chemotherapy; however, the incidence was higher than that reported in large databases for chemotherapy-treated advanced ovary cancer. Thus, these serious adverse events may be direct consequences of the continuous perturbation of DNA repair processes in repair-proficient cells.

Cancer cells demonstrated a higher spontaneous SCE frequency. This elevated rate of genetic instability in cancer cells may potentially promote the acquisition of additional advantageous mutations resulting in more rapid tumor evolution and increasing therapeutic resistance. It will be important to assess the potential for PARPi to promote cancer genome evolution with next-generation sequencing in ongoing clinical trials, although the concern regarding promotion of genetic instability or induction of a mutator phenotype with therapeutics may be inherent, to some degree, in most if not all agents that target DNA or the DNA damage response.

In summary, we have demonstrated genetic instability induced by PARPi at low dose in actively growing cells. Whether short duration therapy may be useful intermittently to eliminate clones of cells in BRCA mutation carriers that have lost the wild-type allele is uncertain. The argument that any exposure to PARPi will cause genome instability in a proliferating cell is supported by our analysis. Most events are not deleterious, including equal SCE that are genetically neutral. However, over 10,000 SSBs are estimated to occur per day in a mammalian cell [43], such that perturbation in their repair and the generation of additional damage confers a significant risk of genetic instability. Hyperrecombination and its associated genetic instability is clearly an acceptable risk in the effective treatment of advanced cancer. Whether it is an acceptable risk for early stage cancer, cancer prevention strategies, and non-oncologic indications needs careful consideration.

## Materials and Methods

### Cells, chemicals and survival assays

Human mammary epithelial cell lines used included non-tumorigenic diploid MCF-10A, HMEC-hTERT and tumorigenic MDA-MB-468 (basal) and MCF-7 (luminal). MCF-10A was provided by B. H. Park (John Hopkins Univ Sch of Med) [44], HMEC-hTERT was provided by A. Hall (Memorial Sloan Kettering Cancer Center (MSKCC)) [45], MDA-MB-468 was provided by Q.B. She (MSKCC) [46], and MCF-7 was provided by S. Powell (MSKCC). Blood samples were obtained from normal donor participants. The participants provided written informed consent to IRB protocol #95–054 approved by the Institutional Review Board at MSKCC. The original signed consent is then sent to the research participant’s medical record and a copy of the consent is given to the participant. The EBV-transformed B-lymphocyte cell line (EBV-BL) was generated by transformation of peripheral blood mononuclear cells (PBMC) with the B95.8 strain of EBV, as previously described [47]. Primary T cells were generated from PBMC from two normal donors by non-specific activation with phytohemagglutinin and cultured in RPMI1640 and human serum AB for 4–14 days before use [47]. E14 mES cell line was provided by M. Hooper [48], *Brca1*^-/-^ 236.44 mES cell line was provided by B. H. Koller [49], *Brca2*^*lex1/lex2*^ mES cell line was provided by P. Hasty and Lexicon Genetics [50]. E14 wild-type, *Brca1*^-/- 236.44^, and *Brca2*^*lex1/lex2*^ mES cell lines were cultured in standard ES media [51]. *Brca1*^-/- 236.44^ cells express a BRCA1 peptide deleted for sequences encoded by exon 11 [52], while *Brca2*^*lex1/lex2*^ cells express a BRCA2 peptide deleted for the terminal exon 27 [53]. Olaparib was synthesized in the MSKCC Organic chemistry core facility. Veliparib and BSI-201 were purchased from Chemie Tek. The compounds were dissolved in DMSO and PBS. For clonogenic survival assays, MCF-10A (1000 cells), HMEC-hTERT (1000 cells), MDA-MB-468 (1500 cells) and mouse ES cell (2000 cells) were seeded, vehicle control or inhibitors were added at the indicated concentration 24 hours after seeding and changed every three days for continuous treatment. At 7–14 days, colonies were fixed with methanol and stained with Giemsa. For EBV-BL (6 × 10^6^) and primary T cells (3.6 × 10^6^) were continuously exposed to the indicated concentration of drug for 10 days at 0.6 × 10^6^ cells/ml. Viable cells were scored by trypan blue exclusion.

### Chromosome analysis

To prepare metaphase spreads, exponentially growing cells were treated with 0.1–0.3 μg/ml of colcemid for 1–6 hr. Cells were collected and incubated in hypotonic solution (0.56% KCl), fixed in methanol: acetic acid (3:1), spotted to slides and air-dried. To measure chromosomal aberrations, the slides were stained with 2% Giemsa/Sorensen’s buffer. To visualize SCEs, the doubling time for each cell type was determined. Cells were incubated with 10 μM BrdU for two cell cycles. After metaphase spreads were prepared, the slides were treated with 0.5 μg/ml Hoechst 33258. The slides were exposed to black light for 20–60 min, incubated at 60°C for 2 hr, and stained in 4–10% Giemsa/Sorensen’s buffer.

### PARP activity assay

Cellular PARP activity was determined by measuring net PAR expression using HT PARP *In vivo* Pharmacodynamic Assay (Trevigen) as specified by the manufacturer. PAR levels in samples were calculated using standard curves and normalized using protein concentration. Protein concentration was measured with Lowry protocol using RC DC Protein Assay Kits (Bio-Rad).

## Supporting Information

S1 Fig Characteristics of human cells. (PDF)

S2 Fig SCEs are higher in cancer cells than in repair proficient non-tumorigenic cells. (DOCX)

S1 Table Characteristics of human cells. (PDF)

S2 Table SCE frequencies of human cells with or without olaparib. (PDF)

S3 Table Chromosomal aberrations of human cells with or without olaparib. (PDF)

S4 Table SCE frequencies of two human normal cell lines with or without PARP inhibitors. (PDF)

S5 Table SCE frequencies with combination exposure. (PDF)

S6 Table Chromatid-type aberrations with combination exposure. (PDF)
