# Supplementary material for: PARP Inhibitors in Clinical Use Induce Genomic Instability in Normal Human Cells
Source: PLoS One. 2016 Jul 18;11(7):e0159341. doi: 10.1371/journal.pone.0159341 (PMC4948780; doi:10.1371/journal.pone.0159341)
Supplement: S1 Fig — (PDF) [file pone.0159341.s001.pdf]

S1 Fig. Characteristics of human cells.

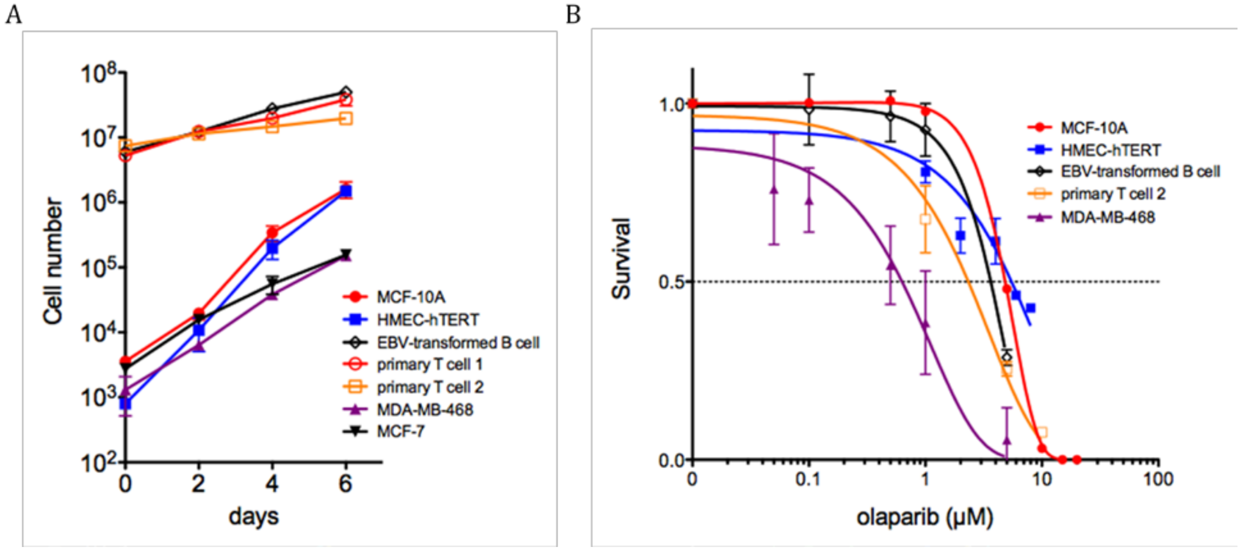

A. Characterization of doubling time for SCE experiments. For adherent cells, cells were incubated in 6-well plate. For lymphocytes, cells were incubated in flasks at 0.6×10^6 cells/ml. Cell proliferation was measured by counting live cells at 48 hr intervals. Means with SD are shown (n = 3).

B. Dose-response survival curves with olaparib. For adherent cells, survival was assessed by clonogenic survival assay. For lymphocytes, viable cells were scored by trypan blue exclusion. Cells were grown with continuous exposure at the indicated concentration of olaparib. Means with SD are shown (n = 3).
