# Supplementary material for: PARP Inhibitors in Clinical Use Induce Genomic Instability in Normal Human Cells
Source: PLoS One. 2016 Jul 18;11(7):e0159341. doi: 10.1371/journal.pone.0159341 (PMC4948780; doi:10.1371/journal.pone.0159341)
Supplement: S2 Fig — (DOCX) [file pone.0159341.s002.docx]

**S2 Fig. SCEs are higher in cancer cells than in repair proficient non-tumorigenic cell**

Spontaneous SCE and olaparib-induced SCE are shown for each cell type. Both spontaneous (A) and olaparib-induced (B) SCEs are significantly higher in tumorigenic cells as compared to non-tumorigenic cell lines and primary cells. Error bars depict means with SD. One asterisk indicates a *P* < 0.0001, unpaired *t*-test.
