# Supplementary material for: PARP Inhibitors in Clinical Use Induce Genomic Instability in Normal Human Cells
Source: PLoS One. 2016 Jul 18;11(7):e0159341. doi: 10.1371/journal.pone.0159341 (PMC4948780; doi:10.1371/journal.pone.0159341)
Supplement: S1 Table — (PDF) [file pone.0159341.s003.pdf]

S1 Table: Characteristics of human cells

Cell type	Doubling time (hr)	IC_{50} of olaparib (μM)
non-tumorigenic		
mammary epithelial cell lines		
MCF-10A	16.0	4.7[a]
HMEC-hTERT	13.4	5.5[a]
lymphocytes		
EBV-BL	52.0	3.6[b]
primary T cell 1	49.9	N/A
primary T cell 2	86.1	2.7[b]
Tumorigenic cell lines		
MDA-MB-468	20.0	0.6[a]
MCF-7	33.7	N/A

[a] clonogenic survival assay

[b] trypan blue exclusion test of cell viability
