# Supplementary material for: PARP Inhibitors in Clinical Use Induce Genomic Instability in Normal Human Cells
Source: PLoS One. 2016 Jul 18;11(7):e0159341. doi: 10.1371/journal.pone.0159341 (PMC4948780; doi:10.1371/journal.pone.0159341)
Supplement: S2 Table — (PDF) [file pone.0159341.s004.pdf]

S2 Table: SCE frequencies of human cells with or without olaparib

cell type	1µM olaparib exposure time (hr)	No. of cells	Mean no. chromosome / metaphase ± SD	Mean no. SCE / metaphase ± SD	Mean no. SCE / chromosome ± SD
MCF-10A	-	51	46.9 ± 1.0	7.6 ± 2.8	0.16 ± 0.06
	30	52	46.9 ± 1.1	73.5 ± 10.9	1.57 ± 0.24
HMEC-hTERT	-	50	46.0 ± 1.4	7.9 ± 2.9	0.17 ± 0.06
	30.5	50	46.0 ± 1.0	59.7 ± 10.0	1.30 ± 0.22
EBV-BL	-	61	46.0 ± 0.4	7.3 ± 3.1	0.16 ± 0.07
	87	52	45.8 ± 0.9	51.6 ± 10.6	1.13 ± 0.23
primary T cell 1	-	55	45.9 ± 0.6	9.5 ± 2.9	0.21 ± 0.06
	99	57	45.8 ± 0.8	41.3 ± 9.3	0.90 ± 0.20
primary T cell 2	-	57	46.1 ± 1.0	8.4 ± 3.4	0.18 ± 0.07
	70	50	45.9 ± 1.0	43.0 ± 9.7	0.94 ± 0.21
MDA-MB-468	-	52	54.4 ± 2.3	13.9 ± 5.0	0.26 ± 0.09
	40	47	54.7 ± 2.0	107.9 ± 21.0	1.97 ± 0.38
MCF-7	-	50	73.4 ± 2.3	22.7 ± 5.0	0.31 ± 0.07
	66.5	50	73.4 ± 2.9	152.4 ± 21.9	2.01 ± 0.30
