# Supplementary material for: PARP Inhibitors in Clinical Use Induce Genomic Instability in Normal Human Cells
Source: PLoS One. 2016 Jul 18;11(7):e0159341. doi: 10.1371/journal.pone.0159341 (PMC4948780; doi:10.1371/journal.pone.0159341)
Supplement: S3 Table — (PDF) [file pone.0159341.s005.pdf]

S3 Table: Chromosomal aberrations of human cells with or without olaparib

cell type	Drug	No. of cells	No. chromosome / metaphase (mean ± SD)	chromatid-type			chromosome-type		total chromatid-type aberrations[a]	total chromatid-type aberrations / metaphase (mean ± SEM)	total chromatid-type aberrations /chromosome (mean ± SEM) (× 10^4)
				gap/ break	radial chromosome	telomere association	gap/ break	dicentric			
MCF-10A	-	100	46.9 ± 0.8	4	0	0	3	0	4	0.04 ± 0.02	8.6 ± 4.2
	+	100	46.8 ± 0.9	20	0	1	3	0	22	0.22 ± 0.05	47.2 ± 9.9
HMEC-hTERT	-	100	46.1 ± 0.8	12	0	0	9	0	12	0.12 ± 0.04	26.4 ± 7.9
	+	100	46.0 ± 1.0	30	0	0	4	0	30	0.30 ± 0.07	65.7 ± 15.8
EBV-BL	-	100	46.0 ± 0.3	24	0	0	2	0	24	0.24 ± 0.05	52.1 ± 10.7
	+	100	46.0 ± 0.6	42	1	0	1	0	44	0.44 ± 0.07	95.5 ± 15.5
primary T cell 1	-	100	45.8 ± 0.5	19	0	0	1	2	19	0.19 ± 0.04	41.5 ± 9.1
	+	100	45.8 ± 0.5	40	2	0	0	3	44	0.44 ± 0.07	95.7 ± 14.9
MDA-MB-468	-	100	54.2 ± 1.1	38	0	1	3	8	40	0.40 ± 0.07	73.9 ± 12.2
	+	100	54.5 ± 1.2	51	5	3	5	4	67	0.67 ± 0.13	123.1 ± 24.8
MCF-7	-	100	73.7 ± 2.5	60	1	0	3	3	62	0.62 ± 0.10	84.3 ± 13.0
	+	100	72.9 ± 2.6	129	4	1	8	4	139	1.39 ± 0.14	191.0 ± 19.9

[a] For calculations of total chromatid-type aberrations, the radial chromosome and telomere association were counted as two aberrations.
