# Supplementary material for: PARP Inhibitors in Clinical Use Induce Genomic Instability in Normal Human Cells
Source: PLoS One. 2016 Jul 18;11(7):e0159341. doi: 10.1371/journal.pone.0159341 (PMC4948780; doi:10.1371/journal.pone.0159341)
Supplement: S4 Table — (PDF) [file pone.0159341.s006.pdf]

S4 Table: SCE frequencies of two human normal cell lines with or without PARP inhibitors

Cell type	Drug	concentration (μM)	No. of cells	Mean no. chromosome / metaphase ± SD	Mean no. SCE / metaphase ± SD	Mean no. SCE / chromosome ± SD
MCF-10A	vehicle control		51	46.9 ± 1.0	7.6 ± 2.8	0.16 ± 0.06
	olaparib	0.1	52	46.9 ± 1.0	33.0 ± 4.9	0.70 ± 0.10
		0.5	53	46.9 ± 0.9	52.2 ± 9.8	1.11 ± 0.21
		1	52	46.9 ± 1.1	73.5 ± 10.9	1.57 ± 0.24
	veliparib	1.5	55	46.8 ± 0.8	26.9 ± 6.6	0.57 ± 0.14
		10	55	46.9 ± 1.0	51.3 ± 9.4	1.09 ± 0.20
		20	57	46.8 ± 1.0	67.0 ± 11.8	1.43 ± 0.25
	BSI-201	20	57	46.8 ± 1.0	7.9 ± 2.9	0.17 ± 0.06
		40	53	46.8 ± 0.9	8.6 ± 2.9	0.18 ± 0.06
		80	55	46.9 ± 0.9	9.4 ± 2.6	0.20 ± 0.06
EBV-BL	vehicle control		61	46.0 ± 0.4	7.3 ± 3.1	0.16 ± 0.07
	olaparib	0.1	56	45.7 ± 0.8	26.0 ± 6.4	0.57 ± 0.14
		0.5	56	45.8 ± 0.6	36.2 ± 7.4	0.79 ± 0.16
		1	52	45.8 ± 0.9	51.6 ± 10.6	1.13 ± 0.23
	veliparib	1.5	54	45.8 ± 0.8	18.7 ± 4.6	0.41 ± 0.10
		10	55	45.9 ± 1.4	32.2 ± 7.2	0.70 ± 0.16
		20	51	45.9 ± 0.5	47.5 ± 11.3	1.04 ± 0.25
	BSI-201	20	54	45.7 ± 0.9	8.4 ± 3.6	0.18 ± 0.08
		40	58	45.8 ± 0.8	9.4 ± 2.9	0.21 ± 0.06
		80	51	45.8 ± 0.9	16.6 ± 4.2	0.36 ± 0.09
