# Supplementary material for: PARP Inhibitors in Clinical Use Induce Genomic Instability in Normal Human Cells
Source: PLoS One. 2016 Jul 18;11(7):e0159341. doi: 10.1371/journal.pone.0159341 (PMC4948780; doi:10.1371/journal.pone.0159341)
Supplement: S5 Table — (PDF) [file pone.0159341.s007.pdf]

S5 Table: SCE frequencies with combination exposure

Cell type	Drug	No. of cells	Mean no. chromosome / metaphase ± SD	Mean no. SCE / metaphase ± SD	Mean no. SCE / chromosome ± SD
MCF-10A	Vehicle control	51	46.9 ± 1.0	7.6 ± 2.8	0.16 ± 0.06
	cisplatin 0.5 µM	53	46.5 ± 0.9	47.7 ± 5.5	1.03 ± 0.12
	olaparib 1 µM	52	46.9 ± 1.1	73.5 ± 10.9	1.57 ± 0.24
	cisplatin 0.5 µM + olaparib 1µM	50	46.5 ± 1.3	90.6 ± 13.0	1.95 ± 0.27
primary T cell 1	Vehicle control	55	45.9 ± 0.6	9.5 ± 2.9	0.21 ± 0.06
	cisplatin 0.5 µM	52	46.1 ± 0.9	36.6 ± 10.8	0.79 ± 0.23
	olaparib 1 µM	57	45.8 ± 0.8	41.3 ± 9.3	0.90 ± 0.20
	cisplatin 0.5 µM + olaparib 1µM	51	46.5 ± 1.3	65.4 ± 11.5	1.41 ± 0.24
