# Supplementary material for: PARP Inhibitors in Clinical Use Induce Genomic Instability in Normal Human Cells
Source: PLoS One. 2016 Jul 18;11(7):e0159341. doi: 10.1371/journal.pone.0159341 (PMC4948780; doi:10.1371/journal.pone.0159341)
Supplement: S6 Table — (PDF) [file pone.0159341.s008.pdf]

S6 Table: Chromatid-type aberrations with combination exposure

cell type	Drug	No. of cells	No. chromosome / metaphase (mean ± SD)	chromatid-type			chromosome-type		total chromatid-type aberrations[a]	total chromatid-type aberrations / metaphase (mean ± SEM)	total chromatid-type aberrations /chromosome (mean ± SEM) (× 10^4)
				gap/ break	radial chromosome	telomere association	gap/ break	dicentric			
primary T cell 1	Vehicle control	100	45.8 ± 0.5	19	0	0	1	2	19	0.19 ± 0.04	41.5 ± 9.1
	cisplatin 0.5 µM	100	46.0 ± 0.5	36	1	0	6	1	38	0.38 ± 0.07	82.4 ± 14.4
	olaparib 1 µM	100	45.8 ± 0.5	40	2	0	0	3	44	0.44 ± 0.07	95.7 ± 14.9
	cisplatin 0.5 µM + olaparib 1µM	100	45.9 ± 0.3	51	3	0	4	0	57	0.57 ± 0.09	123.9 ± 20.6

[a] For calculations of total chromatid-type aberrations, the radial chromosome and telomere association were counted as two aberrations.
